# Gut-directed hypnotherapy in children with irritable bowel syndrome or functional abdominal pain (syndrome): a randomized controlled trial on self exercises at home using CD versus individual therapy by qualified therapists

**DOI:** 10.1186/1471-2431-14-140

**Published:** 2014-06-04

**Authors:** Juliette MTM Rutten, Arine M Vlieger, Carla Frankenhuis, Elvira K George, Michael Groeneweg, Obbe F Norbruis, Walther Tjon a Ten, Herbert Van Wering, Marcel GW Dijkgraaf, Maruschka P Merkus, Marc A Benninga

**Affiliations:** 1 Department of Pediatric Gastroenterology, Emma Children’s Hospital/Academic Medical Center, PO Box 22700, 1100 DD Amsterdam, The Netherlands; 2 Department of Pediatrics, St. Antonius Hospital, Nieuwegein, The Netherlands; 3 Department of Pediatrics, Medical Center Alkmaar, Alkmaar, The Netherlands; 4 Department of Pediatrics, Maasstad Hospital, Rotterdam, The Netherlands; 5 Department of Pediatrics, Isala Clinics, Zwolle, The Netherlands; 6 Department of Pediatrics, Maxima Medical Center, Veldhoven, The Netherlands; 7 Department of Pediatrics, Amphia Hospital, Breda, The Netherlands; 8 Clinical Research Unit, Academic Medical Center, Amsterdam, The Netherlands

**Keywords:** Irritable bowel syndrome (IBS), Functional abdominal pain (FAP), Functional abdominal pain syndrome (FAPS), Hypnotherapy, Children, Pediatrics, Hypnosis, Randomized controlled trial (RCT), Functional gastrointestinal disorders

## Abstract

**Background:**

Irritable bowel syndrome (IBS) and functional abdominal pain (syndrome) (FAP(S)) are common pediatric disorders, characterized by chronic or recurrent abdominal pain. Treatment is challenging, especially in children with persisting symptoms. Gut-directed hypnotherapy (HT) performed by a therapist has been shown to be effective in these children, but is still unavailable to many children due to costs, a lack of qualified child-hypnotherapists and because it requires a significant investment of time by child and parent(s). Home-based hypnotherapy by means of exercises on CD has been shown effective as well, and has potential benefits, such as lower costs and less time investment. The aim of this randomized controlled trial (RCT) is to compare cost-effectiveness of individual HT performed by a qualified therapist with HT by means of CD recorded self-exercises at home in children with IBS or FAP(S).

**Methods/Design:**

260 children, aged 8-18 years with IBS or FAP(S) according to Rome III criteria are included in this currently conducted RCT with a follow-up period of one year. Children are randomized to either 6 sessions of individual HT given by a qualified therapist over a 3-month period or HT through self-exercises at home with CD for 3 months.

The primary outcome is the proportion of patients in which treatment is successful at the end of treatment and after one year follow-up. Treatment success is defined as at least 50% reduction in both abdominal pain frequency and intensity scores. Secondary outcomes include adequate relief, cost-effectiveness and effects of both therapies on depression and anxiety scores, somatization scores, QoL, pain beliefs and coping strategies.

**Discussion:**

If the effectiveness of home-based HT with CD is comparable to, or only slightly lower, than HT by a therapist, this treatment may become an attractive form of therapy in children with IBS or FAP(S), because of its low costs and direct availability.

**Trial registration:**

Dutch Trial Register number NTR2725 (date of registration: 1 February 2011)

## Background

Irritable bowel syndrome (IBS) and functional abdominal pain (syndrome) (FAP(S)) are functional gastrointestinal disorders (FGIDs) that are characterized by chronic or recurrent abdominal pain in absence of an underlying organic disorder causing the symptoms [1]. These disorders affect approximately 20% of children in Western countries and are also prevalent in Asian countries such as Sri Lanka and China, affecting 12,5 to 20% of school-aged children [2-4]. Altered bowel movements and/or relief of abdominal pain after defecation are present in children with IBS, while defecation pattern is normal in children with FAP(S) [1].

Quality of life (QoL) is significantly impaired in most children with IBS or FAP(S): QoL scores are lower compared to healthy peers and comparable to children with inflammatory bowel diseases [5]. Children with IBS or FAP(S) have an increased risk for anxiety and/or depression and have high rates of school absenteeism [6,7]. Health care costs associated with these FGIDs are considerable. Diagnostic workup for a child with IBS or FAP(S) in the United States costs approximately 6000 dollar. The exact costs of treatment of these children are not known, but are also likely to be significant since treatment of adult patients with IBS costs more than $20 billion per year [8,9].

Pathophysiological mechanisms causing IBS and FAP(S) are not fully clarified to this date, but these FGIDs are thought to be a result of a complex interplay of multiple genetic, psychosocial and physiological factors [10]. Standard medical treatment of IBS and FAP(S) consists of a combination of dietary advice, education and pharmacological therapy, such as pain medication, laxatives, anti-diarrheal medication and antispasmodics [11,12]. Despite these treatments, a significant part of children with IBS or FAP(S) continues to experience symptoms, even into adulthood [13].

In the last 30 years, multiple trials have demonstrated that gut-directed hypnotherapy (HT) is an effective therapy in patients with IBS [14-25]. In gut-directed HT, a hypnotic state is induced and a patient is guided to respond to suggestions towards control and normalization of gut functioning, stress reduction and ego-strengthening [26]. Adult IBS-trials have shown that patients receiving HT report significantly lower levels of abdominal pain and symptom scores compared to patients receiving various control treatments. In addition, these effects of gut-directed HT were shown to be long lasting, up to seven years after treatment [20-22]. Gut-directed HT by a therapist has also been studied in children with IBS or FAP(S) and, in accordance with results of adult studies, these trials show HT to be highly effective compared to standard care [23-25] with persisting positive effects after a period of approximately five years [27]. Although these trials show that gut-directed HT is a valuable therapeutic option, especially in children with persisting symptoms, it is still unavailable to many children with IBS or FAP(S), because gut-directed HT performed by a therapist is costly and frequently not reimbursed by health insurance companies. In addition, the number of well-trained child-hypnotherapists is very limited in most countries and the treatment requires a significant investment of both children and parent(s), since visits to the hypnotherapist may cause work and school absences. Van Tilburg et al. studied the efficacy of home-based HT using self-exercises on compact disc (CD) and showed that approximately two thirds of children receiving this home based treatment responded well with >50% reduction in abdominal pain scores [28]. Potential benefits of this treatment at home include that it is less time consuming and less costly compared to HT with a therapist.

Therefore, the primary aim of this study is to compare efficacy of individual HT performed by a qualified therapist with HT by means of CD recorded self-exercises at home in children with IBS or FAP(S). Secondary aims include adequate relief, the cost-effectiveness, effects on depression and anxiety scores, somatization scores, QoL, pain beliefs and coping strategies.

## Methods/Design

### Study design and population

A randomized controlled trial (RCT) with 1 year follow-up is currently conducted to evaluate the efficacy of individual HT performed by a qualified therapist versus HT at home with self-exercises on CD in children with IBS or FAP(S). This study has been designed in line with the methodological recommendations established by the Rome II consensus on ‘Design of treatment trials for gastrointestinal disorders’ [29]. Nine hospitals throughout the Netherlands participate in this RCT. Children are recruited at the outpatient clinic of the department of pediatric gastroenterology of the academic medical centers in Amsterdam and Maastricht and at the outpatient clinic of the departments of pediatrics of the Medical Center Alkmaar, the Flevo Hospital Almere, the Amphia Hospital Breda, St. Antonius Hospital Nieuwegein, the Maasstad Hospital in Rotterdam, the Maxima Medical Center Veldhoven and the Isala Clinics Zwolle. This RCT has been reviewed and approved by the medical ethics committees of all participating hospitals and has been registered in the Dutch Trial Register number NTR2725. It is granted by the Netherlands Organization for Health Research and Development, ZonMW.

A total of 260 children aged 8-18 years diagnosed with IBS, FAP or FAPS according to the Rome III criteria are included in this RCT [1]. Before inclusion, all patients undergo routine laboratory testing to exclude underlying organic disorders: complete blood cell count, C-reactive protein, alanine transaminase (ALT), aspartate aminotransferase (AST), glutamyltransferase (GGT), creatinine, total bilirubin, amylase, celiac screening (anti-transglutaminase antibodies and IgA), urinalysis, stool parasite analysis and *H pylori* antigens in stool. The need for further diagnostic testing is left to the discretion of the treating physician.

Verbal and written information about the study is given by the pediatrician/pediatric gastroenterologist and patients are asked to participate in this RCT if all criteria are met. Subsequently, verbal and written informed consent is obtained from the child and/or both parents. If the child or parent(s) decide not to participate in this RCT, the reason(s) for not participating are documented, if they want to reveal these.

Inclusion and exclusion criteria

Inclusion criteria

(1) Diagnosis of irritable bowel syndrome (IBS) or functional abdominal pain (syndrome) (FAP(S)) according to Rome III criteria [1]

(2) Age 8-18 years at inclusion

Exclusion criteria

(1) Concomitant organic gastrointestinal disease

(2) Treatment by another health care professional for abdominal pain symptoms

(3) Previous hypnotherapy

(4) Mental retardation

(5) Insufficient knowledge of the Dutch language

After obtaining informed consent, children are randomly allocated using a computerized random-number generator for concealment, to either individual hypnotherapy given by a qualified therapist or home-based therapy with hypnotherapy exercises on CD. Randomization is performed on a 1:1 basis with varying block sizes of 2, 4 and 6. Stratification is based on the including hospital and school level (primary/secondary school).

### Interventions

The HT protocol used in this RCT is based on the Manchester protocol for gut-directed HT adapted for children [30] and is comparable to the treatment protocol used in a previous RCT on gut-directed HT in the Netherlands [24]. Hypnotherapy consists of exercises on general relaxation, control of abdominal pain and gut functioning and ego-strengthening suggestions. Several scripts for the exercises used in this RCT are based on scripts used in the van Tilburg et al. trial [28]. Given the nature of HT, blinding of patients and health care professionals involved in the treatment of the participants is not possible.

#### Individual hypnotherapy by a therapist

Individual HT consists of six sessions of 50-60 minutes over a period of three months and is carried out by 11 hypnotherapists affiliated to the recruiting hospitals. All participating hypnotherapists are qualified and have many years of experience in performing HT in children. They have been trained in working with the treatment protocol and instructed to use the same scripts that are used in the CD group, but are allowed to adapt contents and order of these scripts to the child’s interests and specific issues that may come up during therapy. The same protocol is used for children of all ages, but the language used is adapted to the child’s developmental age.

In the first HT session, therapists take a full history. Furthermore, it is explained to both child and parent(s) what hypnotherapy is and how it can help in reducing chronic abdominal pain.

Also, children and parent(s) are instructed not to talk about the pain anymore. In the same session, an exercise on breathing and progressive relaxation is introduced in which children imagine floating on a big cloud. Positive suggestions for decreasing discomfort are given, such as making their hands warm and to place both hands on their belly, imagining the warmth spreading through their abdomen. In the second session, the exercise on progressive relaxation is repeated. Furthermore, an exercise focusing on reduction of anxiety and stress is introduced (‘the favorite place exercise’). In session three, an exercise on ego-strengthening is introduced (‘the rainbow planet exercise’ for children attending primary school; ‘the air balloon exercise’ for children in secondary school). In this hypnosis exercise children choose colors from the rainbow for different needs, for example health, tranquility, courage or confidence. During the fourth session the ‘beach without worries exercise’ is introduced, in which children are encouraged to release stress and again, ego-strengthening suggestions are made. In the fifth session, children do ‘the slide exercise’ in which children visualize sitting on a slide. Suggestions on reduction of anxiety and stress and ego-strengthening suggestions are given as well as visualizations of a well working digestive system with food sliding through the bowel in a comfortable way. In the last session, evaluation of the previous three months will take place. In addition, remaining issues related to the abdominal pain may be addressed and previous exercises are repeated, depending on the needs of the individual patient. Children are advised to keep practicing the HT exercises at home on a regular basis. After the first HT-session all children in the HT-group receive a CD containing standard scripts of all exercises used and they are instructed to listen to the exercises or to practice self-hypnosis on a daily basis. Furthermore, they are encouraged to practice the breathing exercise a few times a day.

#### Hypnotherapy through self-exercises on CD

Children assigned to the CD-group are visited at home by a specially trained research nurse. During this visit the research nurse explains the nature of HT and the exercises to the child and parent(s) and children receive the CD together with an instruction leaflet. In addition, children and parent(s) are instructed not to talk about the abdominal pain anymore. Children are asked to do the first exercise of the CD during this visit, to check whether the child understands the given instructions. The previous trial on home-based treatment has demonstrated that this method is very easy to understand and use [28]. The CD contains five standard scripts of the hypnosis exercises, which are identical to the exercises used by the hypnotherapists. These exercises consist of one exercise on breathing and progressive relaxation and four visualization exercises: ‘the favorite place exercise’, ‘the rainbow planet/air balloon exercise’ (depending on developmental age), ‘the beach without worries exercise’ and ‘the slide exercise’). Two separate CDs are used to make sure that the language used is adapted to the child’s developmental age: one CD for children visiting primary school and one CD for children visiting secondary school. Prior to this RCT, the CD has been tested by three children with chronic abdominal pain and no changes were required. Children are instructed to listen to the hypnosis exercises at least five times a week over a period of three months. Furthermore, they are encouraged to practice the breathing exercise a few times a day. The frequency of listening to the CD will be recorded by the participants in their instruction leaflet. The research nurse will make phone calls to the children after 4 and 8 weeks of treatment to stimulate treatment compliance.

#### Co-interventions

All children participating in this RCT are seen by their pediatrician or pediatric gastroenterologist after three months of HT to evaluate the effects of HT and to provide standard medical care if considered necessary. In addition, children will also visit their physician after 6 months of follow up if necessary.

### Outcomes

Outcomes are measured at baseline (T0), after 3 months of therapy (T1) and at 6 and 12 months follow-up after the end of therapy (T2, T3).

Outcomes are recorded by patients and/or parents at home. At inclusion, a questionnaire on demographics and clinical features is filled out by the treating physician. An overview of the assessments made in this RCT is shown in Table 1.

**Table 1:** Overview of outcomes

**Outcome**	**Instrument**	**T0**	**4 wks**	**8 wks**	**T1**	**T2**	**T3**
Abdominal pain	Abdominal pain dairies	X	X	X	X	X	X
Hypnotic susceptibility	Stanford hypnotic clinical scale for children	X					
Treatment expectations	Self-designed questionnaire for child and parents	X					
Depression and anxiety	Revised Anxiety and Depression Scale-short version (RCADS-25)	X			X	X	X
Somatization	Children’s Somatization Inventory (CSI)	X			X	X	X
Health related quality of life	KIDSCREEN-52	X			X	X	X
Pain beliefs	Pain Beliefs Questionnaire (PBQ)	X			X	X	X
Coping strategies	Children’s Coping Strategies Checklist-revision 1 (CCSC-R1)	X			X	X	X
Cost-effectiveness/cost-utility	Health Utility Index Mark 3 (HUI-3) & costs-questionnaire	X			X	X	X
Adequate relief	Binary question on adequate relief				X	X	X

1. Abdominal pain

The main goal of treatment in IBS/FAP(S) patients is a reduction in levels of abdominal pain.

Abdominal pain is assessed with a diary, in which children record the frequency and intensity of abdominal pain episodes on seven consecutive days [14,24,27,31]. *Pain frequency* is recorded in minutes of abdominal pain per day and is scored as 0 when there was no pain, 1 if children experience 1-30 minutes of pain, 2 for 31-120 minutes of pain and 3 if abdominal pain lasts more than 120 minutes. A pain frequency score (PFS) is subsequently calculated by summing the scores of the seven days, giving a maximum PFS of 21 [24,27,31]. *Pain intensity* is scored using an affective facial scale with faces ranging from showing no pain at all (face A) to the most severe pain (face I). Scores on the facial scale are transported to a daily 0-3 score. No abdominal pain is scored as 0, faces A-C are scored as 1, faces D-F score 2 and faces G-I score as 3. Again, scores of seven days are totaled giving a pain intensity score (PIS), with a maximum of 21 [24,27,31].

The primary outcomes in this RCT are the proportion of patients in which treatment is successful at the end of treatment and the proportion of successfully treated patients after 1 year follow-up. Treatment success is defined as at least 50% reduction in both abdominal pain frequency and intensity scores.

2. Hypnotic susceptibility

Hypnotic susceptibility is defined as a generalized tendency to respond to hypnosis and hypnotic suggestions [32] and is assessed using a Dutch translation of the Stanford hypnotic clinical scale for children [33]. It is administered by the treating hypnotherapist in children assigned to the HT-group and by the research nurse in children assessed to the CD-group. The Stanford hypnotic clinical scale for children consists of seven items. Scores are based on assessment of the child’s behavior and experiences which are verbally reported to the therapist/research nurse. Scores on this scale range from 0 to 7 and higher scores represent higher hypnotic susceptibility.

3. Treatment expectations

Expectations about the response to treatment are assessed for the child, mother and father separately. Child and parents are asked whether they expect the child to improve with treatment and this question is scored on a 11-point scale (0 = not at all; 10 = complete recovery). It is also assessed whether they have a (strong) preference for HT with a therapist or home-based with CD.

4. Depression and anxiety

The short version of the Revised Anxiety and Depression Scale (RCADS-25) is a valid and reliable instrument for the Dutch population to assess symptoms of depression and anxiety [34]. It contains five subscales measuring symptoms of generalized anxiety disorders, separation anxiety disorder, social phobia, panic disorder and major depressive disorder. Each subscale consists of five items, which are scored on a 0 to 3 scale (0 = never; 3 = always). A total score on anxiety is calculated by summing scores on the four individual anxiety scales.

5. Somatization

Child somatization scores are assessed using the Dutch version of the Children’s Somatization Inventory (CSI), which has been shown to be a reliable and valid self-report instrument in children and adolescents [35]. It contains 35 items on the extent to which children experienced somatic symptoms in the previous two weeks. Items are scored on a 5-point scale, ranging from 0 (=not at all) to 4 (=a whole lot). A total score is calculated by summing all 35 individual items and higher scores reflect a higher intensity of somatic complaints experienced by the child. A separate CSI-score for non-gastrointestinal (GI) symptoms can be calculated by leaving out 7 items on GI-complaints: nausea, constipation, diarrhea, epigastric and abdominal pain, vomiting and bloating. To assess GI-complaints other than abdominal pain, items on all GI-symptoms but abdominal pain are summed.

6. Health related quality of life

The KIDSCREEN-52 questionnaire is a frequently used and reliable instrument to measure health related Qol in children and adolescents and has been validated in Dutch pediatric patient groups [36,37]. It contains items on ten dimensions of health related QoL: physical well-being, psychological well-being, moods and emotions, self-perception, autonomy, relations with parents and home life, social support and peers, school environment, social acceptance (bullying) and financial resources. A 5-point Likert scale is used to score each item. Rasch scores for each individual dimension are computed from the individual items and these are transformed into T-values. Higher T-values indicate a better health related QoL and well-being.

7. Pain beliefs

Negative and positive beliefs that children have about their abdominal pain are assessed using the Pain Beliefs Questionnaire (PBQ). A Dutch translation of the PBQ, which is reliable and validated in children and adolescents is used [38,39]. The PBQ consists of 32 items scored on a 5-point Likert scale. Negative beliefs can be divided into five subscales, namely condition frequency, condition duration, condition seriousness, episode specific intensity and episode specific duration. The negative beliefs scale is calculated by summing all 20 items on negative beliefs. The other 12 items assess problem focused coping potential (PFCP) and emotion focused coping potential (EFCP). The PFCP and EFCP scales are computed by averaging the 6 items belonging to both scales. Higher scores on a scale indicate that a child has such thoughts more frequently.

8. Coping strategies

The Dutch version of the Children’s Coping Strategies Checklist-revision 1 (CCSC-R1) is used to measure strategies for coping with everyday problems [40,41]. The CCSC-R1 includes 54 items, all starting with the same phrase ‘if I have a problem…’ and all items are scored on a 4-point Likert-scale (1 = never; 4 = always). This questionnaire has sound psychometric properties and comprises five dimensions: problem focused coping, positive cognitive reframing, distraction strategies, avoidance strategies and support seeking strategies. Scale scores on all five dimensions are calculated by averaging the individual items belonging to the five scales.

9. Cost-effectiveness/cost-utility

The Health Utility Index Mark 3 (HUI-3) is applied as a multi-attribute utility measure of health status and will be used in the cost-effectiveness and cost-utility analysis. It is suitable for assessing children, but because children may be too young to provide reliable and valid information about their own health status, proxy measurements are taken from parents [42-45]. The health utilities derived from the HUI-3 have been anchored with 1 indicating perfect health and 0 indicating death. Quality adjusted life years (QALY) will be calculated by taking the sum of the utility of health states over time by the time in between successive measurements.

In addition to the HUI-3, the Dutch Health and Labor Questionnaire (HLQ) has been adapted to the study setting to measure the direct and indirect costs of health care utilization, work absenteeism by parents, and school absenteeism by children.

10. Adequate relief

Adequate relief has been shown to be a well validated outcome measurement in trials on treatment for IBS [46]. Parents are asked whether their child has adequate relief of IBS-related abdominal pain or discomfort, using a dichotomous scale (yes/no).

### Statistical analysis

Two hundred and sixty children diagnosed with IBS or FAP(S) will be included. The data will be analyzed following the intention to treat principle. The primary analyses will focus on the proportion of patients in both treatment arms in which treatment was successful (>50% reduction abdominal pain frequency and intensity) at T1 and the proportion of patients in which treatment was successful at T3. In addition, abdominal pain levels will be compared in its continuous form using all five moments of measurement during follow-up (after 4 and 8 weeks of treatment, at T1, T2 and T3). These data will be analyzed using linear mixed models to account for correlations of measurements within the same individual. Similar longitudinal, repeated measurement analyses will be performed for the secondary outcomes including depression and anxiety, somatization, health related Qol, pain beliefs, coping strategies and adequate relief. Baseline values will be incorporated in these analyses to adjust for any imbalance at baseline despite randomization and to increase precision by removing between-person variability.

Two explanatory subgroup analyses are planned to evaluate whether there are indications that treatment effects differ between clinical subgroups. Children diagnosed with IBS will be compared to children with FAP(S). Additionally, children in pre-puberty age (13 years or younger) will be compared to older children, since our previous RCT showed that younger children showed a better treatment response up till 6 months after treatment [24].

Economic evaluation will be performed from a societal perspective. Cost-effectiveness and cost-utility ratios will be calculated for the additional costs per extra patient with at least 50% reduction of abdominal pain scores and the additional costs per extra QALY.

#### Power

The primary analysis focuses on the proportion of patients with at least 50% reduction in abdominal pain levels compared to baseline at the end of therapy and after one year of follow up. Based on previous trials, we made a conservative estimate of this percentage being around 75% in the group receiving HT performed by a therapist. In the group assigned to self-hypnosis with CD, we anticipated this percentage to be marginally lower at 65%. In order to be a reasonable alternative treatment, this percentage should not become lower than 50% in the CD group. This non-inferiority margin should be viewed in relation to success rated with standard medical care of around 30-40% in previous trails [23,24,28]. Based on these expected proportions and a non-inferiority margin at 50%, a total of 115 patients per group are needed to achieve a power of 80% with a one-sided significance level of 5%. Since we expect less than 10% drop-out, our aim is to include 130 patients per group.

## Discussion

To our knowledge, this is the first RCT comparing the effectiveness of gut-directed HT performed by a qualified therapist to home-based HT with self exercises in CD in children with IBS or FAP(S). It has been designed according to methodological recommendations on the design of treatment trials for gastrointestinal disorders and has several strengths [29]. A total of 260 children with a Rome III diagnosis of IBS or FAP(S) are included in this RCT, while previous pediatric trials on HT had sample sizes up to 52 patients [23-25,28]. Generalizability of the results of this trial will be increased, because we include both younger children and adolescents, patients from urban and rural areas in the Netherlands and recruit children from both academic centers and teaching hospitals. In addition, children are followed for a period of 1 year after the end of therapy, to evaluate whether initial effects are sustained over time. In contrast with our earlier HT trial, in which all children were treated by the same hypnotherapist [24], in this study eleven different hypnotherapists are involved in the treatment of children with abdominal pain. This will shed light on the influence of the therapist with regards to efficacy. By assessing secondary outcomes, such as depression, anxiety and somatization, it may become possible to predict response to HT, thereby allowing clinicians to select children that are likely to benefit from HT. A possible limitation of this study may be the fact that children and parents are not blinded for the received treatment, which is not possible due to the nature of HT. Recording of outcome measures by children and parents themselves at home, instead of recording of outcomes by a health care professional during a visit to the hospital, however, reduces the risk of detection bias. In addition, the fact that symptoms of abdominal pain are recorded for seven consecutive days has the benefit that it corrects for individual variability of symptoms over time. Response expectancies are known to (partly) mediate effects of psychological treatments, such as HT [47]. We therefore record expectancies about HT from the child and both parents separately to assess the magnitude of expectation bias. Another possible limitation of the design of this RCT may be the fact that we did not include a third treatment arm with children receiving standard medical care. This third treatment group however was waived, since reviewers considered it being not ethical to abstain children from treatment with HT, because previous studies have shown HT to be superior to standard medical care in this group of patients [23,24,28].

If this RCT will show that the effectiveness of home-based HT with CD is comparable to or only slightly lower than HT by a therapist, this treatment may become an attractive first line of treatment in children with IBS or FAP(S). Since it presumably is less costly and less time-consuming, it will benefit children, parents and society. In addition, the large number of children with IBS and FAP(S) combined with a shortage of qualified therapists, causes long waiting lists nowadays. Home-based HT can be started as soon as the diagnosis of IBS or FAP(S) has been made, without dealing with these long waiting lists. A potential scenario after this RCT might be that a significant proportion of children with IBS or FAP(S) can get this home-based treatment, prescribed by their general practitioner. This may subsequently lead to fewer referrals to pediatricians and/or pediatric gastroenterologists, which would lead to an additional decrease in health care costs. If this RCT indeed shows these results, the next step will be implementation of home-based HT into clinical care for children with IBS or FAP(S).

## Abbreviations

CD: Compact disc; FAP: Functional abdominal pain; FAPS: Functional abdominal pain syndrome; GI: Gastrointestinal; HT: Hypnotherapy; IBS: Irritable bowel syndrome; PFS: Pain frequency score; PIS: Pain intensity score; QALY: Quality adjusted life years; QoL: Quality of life; RCT: Randomized controlled trial.

## Competing interests

The authors declare that they have no competing interests.

## Authors’ contributions

JR is primary investigator and responsible for data collection, analysis and drafting the manuscript. Participated in the design of the study and contributed to developing of the research protocols. AV supervises the study and participated in the design of the study and contributed to developing of the research protocols. Supervised drafting of the manuscript and critically reviewed it. CF participated in the design of the study, contributed to developing of the research protocols en critically reviewed the manuscript. EG participated in the design of the study, contributed to developing of the research protocols en critically reviewed the manuscript. MG participated in the design of the study, contributed to developing of the research protocols en critically reviewed the manuscript. ON participated in the design of the study, contributed to developing of the research protocols en critically reviewed the manuscript. WT participated in the design of the study, contributed to developing of the research protocols en critically reviewed the manuscript. HW participated in the design of the study, contributed to developing of the research protocols en critically reviewed the manuscript. MD participated in the design of the study, contributed to developing of the research protocols en critically reviewed the manuscript. MM participated in the design of the study, contributed to developing of the research protocols en critically reviewed the manuscript. MB supervises the study, participated in the design of the study and contributed to developing of the research protocols. Supervised drafting of the manuscript and critically reviewed it. All authors read and approved the final manuscript as submitted.

## Pre-publication history

The pre-publication history for this paper can be accessed here:

http://www.biomedcentral.com/1471-2431/14/140/prepub
